# Effects of multiple stressors on river biofilms depend on the time scale

**DOI:** 10.1038/s41598-019-52320-4

**Published:** 2019-11-01

**Authors:** Ferran Romero, Vicenç Acuña, Carme Font, Anna Freixa, Sergi Sabater

**Affiliations:** 1 Catalan Institute for Water Research (ICRA), C. Emili Grahit 101, 17003 Girona, Spain; 2 Universitat de Girona (UdG), Girona, Spain; 3 Institute of Aquatic Ecology (IEA), University of Girona, Campus de Montilivi, 17003 Girona, Spain

**Keywords:** Environmental impact, Water microbiology, Environmental impact, Water microbiology

## Abstract

Global change exposes ecosystems to a myriad of stressors differing in their spatial (i.e. surface of stressed area) and temporal (i.e. exposure time) components. Among freshwater ecosystems, rivers and streams are subject to physical, chemical and biological stressors, which interact with each other and might produce diverging effects depending on exposure time. We conducted a manipulative experiment using 24 artificial streams to examine the individual and combined effects of warming (1.6 °C increase in water temperature), hydrological stress (simulated low-flow situation) and chemical stress caused by pesticide exposure (15.1–156.7 ng L^−1^) on river biofilms. We examined whether co-occurring stressors could lead to non-additive effects, and if these differed at two different exposure times. Specifically, structural and functional biofilm responses were assessed after 48 hours (short-term effects) and after 30 days (long-term effects) of exposure. Hydrological stress caused strong negative impacts on river biofilms, whereas effects of warming and pesticide exposure were less intense, although increasing on the long term. Most stressor combinations (71%) resulted in non-significant interactions, suggesting overall additive effects, but some non-additive interactions also occurred. Among non-additive interactions, 59% were classified as antagonisms after short-term exposure to the different stressor combinations, rising to 86% at long term. Our results indicate that a 30-day exposure period to multiple stressors increases the frequency of antagonistic interactions compared to a 48-hour exposure to the same conditions. Overall, the impacts of multiple-stressor occurrences appear to be hardly predictable from individual effects, highlighting the need to consider temporal components such as duration when predicting the effects of multiple stressors.

## Introduction

Freshwater ecosystems are currently threatened by global pressures on land use and climate, affecting ecosystem stability and biodiversity^1^. Among freshwater ecosystems, rivers and streams are particularly vulnerable to stressors derived from land-use and climate change, and multiple stress occurrences have been identified as responsible for river biodiversity loss^2^. However, these effects are difficult to predict because of the complexity of the interactions between stressors^2–4^. Multiple stressors may interact in additive or in complex (non-additive) ways, where the responses of the combined effects of multiple stressors may be greater (synergistic) or smaller (antagonistic) than what would be predicted based on the individual stressor effects involved^5^. Recent analyses have emphasized that interactions in river ecosystems may account for 40% to 69% of all ecological responses^2,3,6^ and that non-additive interactions may be as frequent as additive responses^4^, indicating that multiple stressor effects are hard to predict based on effects attributed to single stressors. A recent literature review suggested that the differences observed may depend on the type of ecosystem and the organization level studied, from individual species to populations and whole ecosystems^7^. Understanding these often overlooked multiple-stressor effects is still seen today as one of the most pressing challenges in ecology.

Global change and its associated stressors, such as warming, river flow reductions and chemical exposure due to land-use changes are particularly urgent issues in riverine areas. The Intergovernmental Panel on Climate Change (IPCC) indicates that greenhouse gases emissions will increase global temperatures between 1.5 °C and 4.5 °C before 2050^8^. Combined with the expected lower-than-average precipitation events, this could suppose a reduction in river flow of −16% to −35% compared to pre-industrial periods in areas already suffering from limited water resources such as the Mediterranean region^9^, with potential implications for habitat conditions and biodiversity^10^. Global environmental change also affects land uses associated to high urbanization^11^ and increasing demand for food production^12^, thus shifting natural land use from forest to agricultural fields^13^. Streams and rivers draining agricultural catchments are highly impacted by elevated levels of dissolved nutrients^14^, deposited fine sediments^15^ and pesticides^16,17^. Thus, climate and land-use changes force multiple stress scenarios onto river ecosystems, which may produce uncertain outcomes.

River biota is directly impacted by multiple stressors. Amongst river and stream organisms, river biofilms play a key role in nutrient processing and river functioning^18^. Biofilms occupy different habitats on the riverbed, which favor the occurrence of compositional variability and complexity^19^. Biofilms developing on hard river surfaces (cobbles and rocks) are known as *epilithic biofilms*. When dissolved nutrients are not limiting and light reaches the riverbed, epilithic biofilms are usually dominated by primary producers (algae, cyanobacteria), whereas under light limitation as it might occur in small streams with dense canopies, heterotrophs become more important^20^ Conversely, biofilms that develop on sub-superficial fine sediments (e.g. sand) are known as *epipsammic biofilms*, and are mostly composed by heterotrophic microorganisms, such as bacteria and fungi. Because of the higher porosity of fine sediments, epipsammic biofilms are less affected than epilithic biofilms by hydrological stress^21^. The different composition and attributes of epilithic and epipsammic biofilms may be involved in their specific response to single stressors^22,23^, differing both on the velocity of response as well as in the degree of tolerance^24–28^.

Several studies have already assessed the short-term (i.e. hours) effects of multiple interacting stressors on river biofilm communities^26,28^. Others have also shown that effects can appear in the long term^29–31^. The photosynthetic efficiency of algae and cyanobacteria and the enzymatic activities of heterotrophs become rapidly altered after river biofilm exposition to herbicides^28^, or to physical stressors such as warming or hydrological stress^26,27^. There are indications that responses might differ according to the exposure time; a sustained stress can promote changes in a community, selecting the most resistant species^32^ and therefore favoring community adaptation to the new conditions^33^. In long-term exposures (e.g. weeks), ecosystem function may experience pronounced shifts^34^, which usually come along with structural changes^24,25^. Previous work with river biofilms has demonstrated that extended non-flow periods promote changes in the production-respiration ratios in biofilm communities, leading it towards heterotrophy^35^. Thus, there is a need to produce experimental designs focusing on multiple stressor effects at different time scales, and including several structural and functional descriptors.

This study aimed to evaluate the individual and interactive effects of three stressors (namely hydrological stress, warming and a pesticide mixture) on river biofilms at two different time scales (i.e. after 48 hours and 30 days of stressor exposure). To do so, a full-factorial design (2^3^) was used and river biofilms were exposed to either individual or combined stressors. We could therefore produce an experimental design focused on the effect of exposure time on the size and direction of the interactive stressor effects. We hypothesized that (i) stressors associated to climate change (warming and hydrological stress) would cause the most pervasive effects, as they encompass multiple level effects derived from hindered resource acquisition and overall physiological disruption and (ii) antagonism would be the main non-additive interaction type, particularly in the long term, because of the high potential for adaptation of the biofilm community to stressors.

## Materials and Methods

### Experimental design

The experiment was performed at the indoor Experimental Streams Facility of the Catalan Institute for Water Research (Girona, EU), between July 3^rd^ and August 22^nd^, 2017. Each of the 24 artificial streams (see next section, 2.2) was assigned one of seven experimental treatments (W, warming; H, hydrological stress; P, pesticides; W:H, W:P, H:P and W:H:P), or controls (C), following a full-factorial replicated (n = 3) design with 3 fixed factors (i.e. W, H, P) and 2 levels per factor (i.e. presence vs. absence of the stressor). We also included exposure time (T) and substratum type (S) in our analyses, as they were treated as random factors (see statistical analyses, section 2.5). River sediment was transported from an unpolluted reference site (see next section, 2.2) and allowed to acclimate under control conditions for 16 days. After the acclimation period, treatments were applied for 35 days. All the response variables were assessed after 48 hours (short-term effects) and after 30 days (long-term effects) of treatment exposure.

### Experimental conditions

Each artificial stream consisted of an independent methacrylate channel (l – w – d = 200 cm–10 cm–10 cm) and a 70 L water tank from which water could be recirculated (Fig. S1). Each artificial stream was filled with 5 L of fine sediment (i.e. sand) extracted from an unpolluted segment of the Llémena River (GPS WGS84; 42°04′03.6″N, 2°36′34.1″E, Sant Esteve de Llémena, Girona, EU), which is a permanent river draining a calcareous mountainous range (d_50_ = 0.74 mm). The extracted sand was transported in less than one hour to the artificial streams, and then evenly distributed to create a plane bed covering the bottom of the streams. At complete water saturation, the porosity of the sand yielded a water content of 25% of the wet weight. The sand was used to colonize the epipsammic biofilm, whereas, in order to assess the response of the epilithic biofilm, small flat cobbles (mean surface = 42.5 ± 11.0 cm^2^) were extracted from the Llémena River, transported to the laboratory and distributed on the streams. Each stream received a constant flow of 60 mL s^−1^ from the tank, and operated as a closed system for 72 h, as water from all the streams was renovated every three days. Mean water velocity was 2 cm s^−1^, and water depth over the plane bed was 3 cm. Daily cycles of photosynthetic active radiation (PAR) were defined as 10 h daylight + 14 h darkness and were simulated by LED lights (Lightech, Girona, EU). PAR was held constant at 173.99 ± 33 μE m^−2^ s^−1^ during the daytime, and was recorded every 10 min using 4 quantum sensors located across the whole array of streams (sensor LI-192SA, LiCOR Inc, Lincoln, USA). Air temperature was maintained at 15 °C during the acclimation period and at 20 °C during the exposure period, at a constant air humidity of 30%. Water temperature was recorded every 10 min using VEMCO Minilog (TR model, AMIRIX Systems Inc, Halifax, NS, Canada) temperature data loggers (−5 to 35 °C, ±0.2 °C).

All treatments were applied simultaneously after the 16-day acclimation period. Accordingly, Cryo-Compact Circulators (Julabo CF-31, Seelbach, Germany) were used to achieve an average water temperature increase of 2 °C in all the treatments including warming as a stressor. Hydrological stress was applied by reducing the water flow from 60 to 5 mL s^−1^, for which the fine sediments covering the bottom of the artificial streams remained slightly wet, while the cobbles became completely desiccated. Pesticide exposure consisted of a mixture composed by two herbicides (i.e. Diuron and Simazine), two fungicides (i.e. Imazalil and Prochloraz) and one insecticide (i.e. Chlorpyrifos). Nominal concentrations of each compound in the mixture are presented in Table S1. All the used compounds were purchased by Sigma-Aldrich. The mixture of pesticides was freshly prepared in each water renewal (each 2–3 days) at a concentration of 100 mg L^−1^ in 50% methanol: water (v: v). The total concentration of methanol reaching the artificial streams was 400 ng L^−1^, representing 0.0005% of the total water volume. The same concentration of methanol was added in the pesticide-free controls. The mixture of pesticides was added using peristaltic pumps (IPC Microprocessor pump, IDEX Health & Science GmbH_Ismatec, Switzerland). The compounds included in the mixture and their nominal concentrations were selected because of their common occurrence and frequency in rivers draining agricultural catchments (see Table S2 for references).

### Water physical and chemical properties

#### General descriptors

Dissolved oxygen, pH and specific conductivity were measured in each artificial stream using WTW (Weilheim, Germany) hand-held probes. Nutrient and dissolved organic matter concentrations were measured from the water collected from the stream outlet. Both physical and chemical parameters were measured after short (i.e. 48 h) and long-term (30 d) exposure to the experimental treatments in 12 randomly selected streams (out of a total 24). Water was filtered immediately through 0.2 μm pore nylon filters (Whatman, Kent, UK) into pre-washed polyethylene containers for nutrient analyses and through 0.7 µm glass fiber filters for DOC analyses. Detection and quantification of nutrients and DOC were performed according to standard procedures previously used in the Experimental Streams Facility^36^.

#### Pesticide quantification

All standards for the target compounds were obtained from Sigma-Aldrich. Stock solutions of the pesticides Diuron, Imazalil, Prochloraz, Simazine and Chlorpyrifos were prepared from powder in methanol at 1 mg mL^−1^, which was stored frozen at −20 °C. Chemical information and nominal concentrations of the pesticides are available in Table S1. Working standard solutions as well as the calibration standard curve were prepared by appropriate dilution in methanol:water (10:90, v-v) of the stock solution. Water samples for pesticide analyses (1000 mL) were collected 48 hours after the beginning of the experimental manipulation phase (short-term exposure) and after 30 days (long-term exposure) from all artificial streams. The collected samples were filtered through 0.45 μm polyvinylidene fluoride membrane filters (PVDF, Millipore) and analyzed using ultra-performance liquid chromatography (UPLC, Waters Corp. Milford, MA, USA) coupled to an hybrid quadrupole-linear ion trap mass spectrometer (5500 QTRAP, Applied Biosystems, Foster City, USA) (LC-MS/ MS system).

### Sampling and sample processing

A total of 11 biofilm variables (chlorophyll-*a* concentration, photosynthetic efficiency, photosynthetic capacity, chlorophyll basal fluorescence, leucine aminopeptidase activity, substrate utilization richness and diversity, 16S rRNA gene abundance, gross primary production, community respiration and production-respiration ratio) were measured after short (48 h) as well as long-term (30 days) exposure to stress conditions. Metabolic rates (i.e. community respiration and gross primary production) were measured from wire net baskets containing epilithic and epipsammic biofilm (see section 2.4.6); all the other variables were measured separately from epilithic and epipsammic biofilms. Photosynthetic efficiency, photosynthetic capacity and chlorophyll-*a* basal fluorescence were measured *in-situ* using a Diving PAM (Pulse Amplitude Modulated) underwater fluorometer^37^ (Heinz Wlaz, Effeltrich, Germany, see section 2.4.2). Chlorophyll-*a* concentration, Leucine aminopeptidase activity, substrate utilization and 16S rRNA gene abundance were measured from re-suspended biofilm in filtered (0.2 μm) stream water (see sections 2.4.1, 2.4.3, 2.4.4 and 2.4.5).

#### Algal biomass determination

Chlorophyll-*a* concentration was used to evaluate biofilm structural changes after a 90% acetone extraction, done overnight in dark conditions at 4 °C; it was quantified spectrophotometrically using a Lambda UV/VIS spectrophotometer (U-2000 Spectrophotometer; Hitachi, Tokyo, Japan). Chlorophyll-*a* concentration is expressed in μg·cm^−2^.

#### *In vivo* fluorescence measurements

Biofilms were analyzed *in-vivo* to determine three chlorophyll fluorescence-derived parameters; namely photosynthetic efficiency (Y_eff_), photosynthetic capacity (Y_max_) and chlorophyll basal fluorescence (F_0_) using a Diving PAM (Pulse Amplitude Modulated) underwater fluorometer (Heinz Wlaz, Effeltrich, Germany). Y_eff_ was determined under steady-state conditions, whereas F_0_ and Y_max_ were measured after a 30-min adaptation to dark conditions. Y_eff_ and Y_max_ indicate the fraction of light that is converted into chemical energy during photosynthesis, and can therefore be used as a measure to evaluate functional changes in the algal component of the biofilm after exposure to environmental disturbances^37^.

#### Leucine aminopeptidase activity

The degradation capacity of peptides was assessed by measuring the activity of the extracellular enzyme leucine aminopeptidase (LAP). LAP was here used as a functional parameter to assess the capacity of the bacterial compartment to degrade peptidic compounds. It was measured using fluorescent-linked substrata (aminomethyl-coumarin, AMC). Biofilms were incubated for 1 h in the dark at 12.5 °C immediately after collection. Blanks and standards of AMC (0–100 μmol L^−1^) were also incubated. At the end of the incubation, a glycine buffer (pH 10.4) was added (1/1 vol/vol), and fluorescence was measured at 364/445 nm excitation/emission for AMC. Values were expressed as nmol of released AMC cm^−2^ h^−1^.

#### Organic substrate utilization (biolog ecoplates)

Biolog Ecoplates (Biolog Inc. Hayward, California, USA) were used to assess the differences in the substrate utilization capacity of different biofilm samples. Each Biolog Ecoplate contains three replicated wells of 31 different carbon sources and a blank with no substrate. Biofilms were extracted and diluted using a Ringer solution (1:20). Then, Ecoplates were inoculated with 130 µL of biofilm extract, under sterile conditions and incubated at 20 °C in the dark. Plates were read every 24 h until an asymptote was reached, which took between 6 and 7 days at 590 nm using a microplate reader (Epoch microplate reader, Biotek instruments, Winooski, USA). Data treatment followed the procedure described in ref.^38^. Briefly, raw absorbance data for each well was corrected by taking away the mean absorbance of the control wells (without substrate) and negative values, as well as low absorbance values (<0.05) were set to zero. Finally, Shannon diversity index and substrate richness (i.e. the number of positive wells) were calculated using data from wells when the Average Well Colour Development was closest to 0.5^39^.

#### Abundance of 16S rRNA gene copies

The 16S rRNA gene was used to assess the overall structural response of the bacterial community to the treatments^40,41^. Extraction of DNA was performed on samples of 200 mg of freshly detached biofilm using the FastDNA® SPIN kit for soils (MP Biomedicals) following manufacturer instructions. DNA concentration in each sample was measured using Qubit 2.0 fluorometer (Life Technologies; Carlsbad, CA, USA); its purity was determined by measuring A260/A230 and A260/A280 absorbance ratios using a NanoDrop 2000 spectrophotometer (Thermo Fisher Scientific; Wilmington, USA). Standard quantitative PCR (qPCR) procedure was used to quantify abundance of 16S rRNA gene on DNA extracted from epilithic and epipsammic biofilms. Quantitative PCR conditions are detailed in ref.^41^.

#### Metabolic biofilm rates

Metabolic biofilm rates were assessed through changes in oxygen concentration (oxygen balance method) under light and dark conditions. Trays containing 34 cm^2^ of fine sediment and one cobble from each artificial stream were removed and incubated in cylindrical acrylic chambers (volume 0.96 L). Each chamber was provided with a submersible water circulation pump to avoid the formation of zones of low diffusion within the chamber. The incubations for each metabolic rate (net primary production and community respiration) lasted for 45 min, and were carried out inside an incubator chamber (Radiber AGP-700-ESP, Barcelona, Spain) at the same temperature and light conditions than those of the artificial streams. Net primary production was measured under light conditions, and community respiration was measured in the dark. Dissolved oxygen concentration inside the chambers was measured continuously with oxygen sensors and logged at 15 s intervals (PreSens OXY-10 mini, Regensburg, Germany). Gross primary production and community respiration were calculated according to ref.^42^.

### Statistical approach

We examined the response of the different community-level metrics on epilithic and epipsammic biofilms, as well as the overall metabolic biofilm response. For each metric, we ran a mixed-model nested ANOVA with the factors *warming* (W; fixed factor, 2 levels), *hydrological stress* (H; fixed factor, 2 levels), *pesticides* (P; fixed factor, 2 levels), *time* (T; random factor, nested in W, H and P, 2 levels) and *substratum type* (S; random factor, nested in W, H and P, 2 levels). Within the ANOVA results, we obtained F-ratios as the division of the Mean Sum of Squares (MSS) among groups and the MSS within groups (residuals). Here, larger F-ratio values may lead to larger treatment effect relative to the within-square error. The interpretation of this statistic depends both on the degrees of freedom of the MSS among and within groups, indicated in lower case. ANOVA was carried out on univariate data using the *aov* function of the package *stats* on R^43^. Two different response patterns where derived from the ANOVA results: *main effects* evaluated the mean performance in the treatments where a given stressor is present, as opposed to the treatments without the stressor. *Interactive effects* were used to evaluate whether the response of a given biofilm metric to the presence of one stressor changed at different levels of additional stressors. Significant interactive effects (ANOVA interaction term P-value < 0.05) were classified into antagonism and synergism according to ref.^3^. Accordingly, antagonism was assumed for stressor combinations resulting in responses less pronounced than predicted from additive effects, whereas synergism was assumed when the opposite pattern was observed (i.e. combined effects amplifying individual effects).

## Results

### Physical-chemical parameters

Water temperature in the artificial streams averaged 18.5 ± 0.4 °C during the acclimation period. After experimental manipulation, it increased to 20.2 ± 0.1 °C in the artificial streams containing *warming* (W) as a stressor (i.e. n = 12; W, W:H, W:P, W:H:P), representing a 1.6 °C increase in water temperature. In the treatments where warming was not a stressor, water temperature averaged 18.5 ± 0.2 °C (n = 12). Added pesticides concentrations ranged from 15.1 to 156.7 ng L^−1^ in the treatments containing *pesticides* (P) as a stressor (i.e. n = 12; P, H:P, W:P, W:H:P). Unexpectedly, chlorpyrifos concentrations were below the detection limit in both the short and long-term measurements (Table 1). Pesticide contamination did not occur in the other artificial streams (n = 12, i.e. C, W, H and W:H, data not shown).

**Table 1:** Physico-chemical characteristics (mean ± S.E, n = 12) of water in 12 randomly sampled artificial streams after 48 hours (i.e. short-term effects) and 30 days of exposure (i.e. long-term effects).

	Short term	Long term
Dissolved oxygen (mg L^−1^)	9.23 ± 0.08	9.02 ± 0.11
Conductivity (µS cm^−1^)	231 ± 1.58	281 ± 8.75
pH	8.90 ± 0.03	8.37 ± 0.11
NO_2_^−^ (mg N- NO_2_^−^ L^−1^)	0.003 ± 0.001	0.003 ± 0.001
NO_3_^−^ (mg N- NO_3_^−^ L^−1^)	1.49 ± 0.06	1.37 ± 0.06
PO_4_^3−^ (mg P- PO_4_^3−^ L^−1^)	0.003 ± 0.001	0.003 ± 0.001
NH_4_^+^ (mg N- NH_4_^+^ L^−1^)	<LOQ^a^	<LOQ^a^
DOC (mg L^−1^)	2.84 ± 0.09	2.30 ± 0.03
Diuron (ng L^−1^)	156.7 ± 51.4	140.7 ± 3.9
Chlorpyrifos (ng L^−1^)	<LOD^b^	<LOD^b^
Imazalil (ng L^−1^)	15.1 ± 2.1	85.4 ± 3.1
Prochloraz (ng L^−1^)	<LOQ^c^	34.2 ± 1.0
Simazine (ng L^−1^)	50.3 ± 2.4	68.6 ± 2.1

Pesticide concentrations correspond only to water samples from contaminated streams (i.e. n = 12, treatments P, W*P, H*P and W*H*P). ^a^The limit of quantification (LOQ) for NH_4_^+^ was 0.001 mg N-NH_4_^+^ L^−1^. ^b^The limit of detection (LOD) for Chlorpyrifos was 2.24 ng L^−1^. ^c^The limit of quantification (LOQ) for Prochloraz was 0.07 ng L^−1^.

### Biofilm responses to warming (W), hydrological stress (H) and pesticides (P)

#### Temporal variation

**Figure 1:**
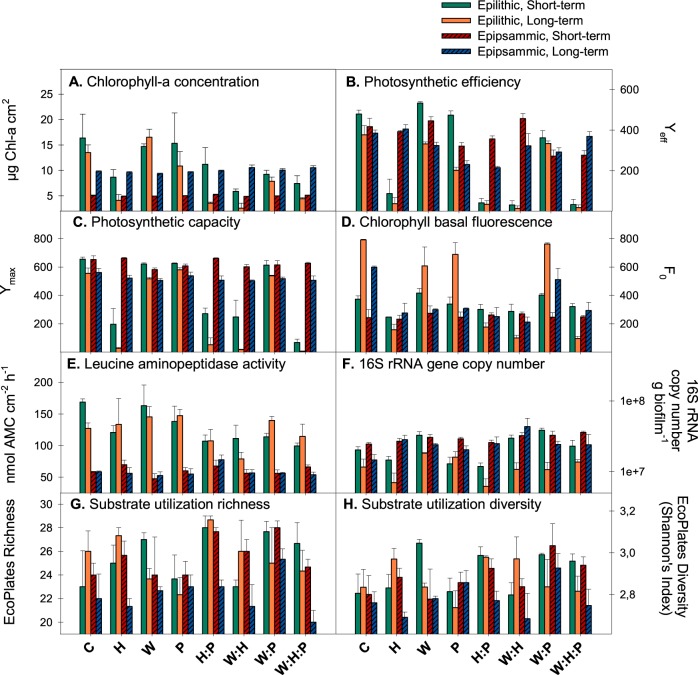
Changes in response variables for epilithic (smooth bars) and epipsammic (stripped bars) river biofilms after short and long-term exposure to the different treatments (hydrological stress; H, warming; W, pesticides; P, H:P, W:H, W:P, W:H:P) and control biofilms (**C**). Plots represent averaged values of chlorophyll-a concentration (**A**), photosynthetic efficiency (**B**), photosynthetic capacity (**C**), chlorophyll basal fluorescence (**D**), leucine aminopeptidase activity (**E**), 16S rRNA gene copy number (**F**), and substrate utilization richness (**G**) and diversity (**H**). Error bars show standard errors between replicates (n = 3).

**Figure 2:**
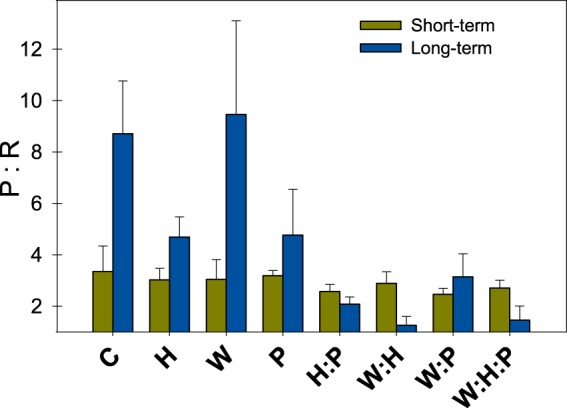
Changes in production-respiration ratios for river biofilms after short and long-term exposure to the different treatments (hydrological stress; H, warming; W, pesticides; P, H:P, W:H, W:P, W:H:P) and control biofilms (C). Bars represent averaged values (n = 3), error bars represent standard errors.

Biofilms in control artificial streams (containing biofilm without stressor addition; n = 3) progressively increased their algal biomass (Figs 1 and 2, Table 2), as suggested by the increasing chlorophyll basal fluorescence (Fig. 1D, Table 2). This increase in chlorophyll fluorescence was only translated into increased total chlorophyll-*a* concentration in the epipsammic biofilm (Fig. 1A; significant interaction between *time* and *substratum type* (S:T)*;* F_1,16_ = 32.3, P < 0.001). Conversely, the number of 16S rRNA gene copies (targeting total bacteria) decreased (factor *time*; F_1,16_ = 32.1, P < 0.001), especially in the epilithic biofilm (Fig. 1F; significant interaction between *time* and *substratum type;* F_1,16_ = 7.6, P = 0.013). Leucine aminopeptidase activity decreased with time in the epilithic biofilm only (Fig. 1E; S:T, F_1,16_ = 16.5 P < 0.001). Overall, the shift towards algal biomass translated into increased production-respiration ratios (Fig. 2) in the biofilms (significant effect of *time*; F_1,16_ = 18.80, P < 0.001).

**Table 2:** Output for the mixed-model nested ANOVA (fixed factors).

Response variable	Factor	df	SS	MS	F	P
Chlorophyll-*a* concentration	**Hydrological stress (H)**	**1**	**2.81E + 02**	**2.81E + 02**	**57.6**	**<0.001**
	H:P	1	5.13E + 01	5.13E + 01	10.5	0.005
Photosynthetic efficiency	**Hydrological stress (H)**	**1**	**6.76E + 05**	**6.76E + 05**	**249.4**	**<0.001**
	Pesticides (P)	1	1.37E + 05	1.37E + 05	50.7	**<**0.001
	H:P	1	1.51E + 04	1.51E + 04	5.6	0.031
Photosynthetic capacity	Warming (W)	1	3.49E + 04	3.49E + 04	8.6	0.010
	**Hydrological stress (H)**	**1**	**2.48E + 05**	**2.48E + 05**	**61.1**	**<0.001**
Basal chlorophyll fluorescence	**Hydrological stress (H)**	**1**	**1.07E + 06**	**1.07E + 06**	**107.4**	**<0.001**
	W:P	1	5.42E + 04	5.42E + 04	5.5	0.033
	W:H:P	1	5.09E + 04	5.09E + 04	5.1	0.038
Leucine aminopeptidase activity	**Hydrological stress (H)**	**1**	**1.33E + 04**	**1.33E + 04**	**10.2**	**0.006**
16S rRNA gene abundance	**Warming (W)**	**1**	**1.56E + 15**	**1.56E + 15**	**33.7**	**<0.001**
Substrate utilization richness	**W:H**	**1**	**8.44E + 01**	**8.44E + 01**	**10.0**	**0.006**
Gross primary production	Warming (W)	1	3.42E + 03	3.42E + 03	18.7	**<**0.001
	**Hydrological stress (H)**	**1**	**5.52E + 03**	**5.52E + 03**	**30.1**	**<0.001**
	Pesticides (P)	1	1.08E + 03	1.08E + 03	5.9	0.027
	W:P	1	1.50E + 03	1.50E + 03	8.2	0.011
	H:P	1	2.53E + 03	2.53E + 03	13.8	0.002
Community respiration	**Warming (W)**	**1**	**4.15E + 03**	**4.15E + 03**	**41.3**	**<0.001**
	Hydrological stress (H)	1	1.65E + 03	1.65E + 03	16.5	**<**0.001
	Pesticides (P)	1	1.63E + 03	1.63E + 03	16.2	**<**0.001
	W:H	1	6.01E-01	6.01E-01	6.0	0.026
	H:P	1	7.93E-01	7.93E-01	7.9	0.013
	W:H:P	1	8.09E-01	8.09E-01	8.1	0.012
Production-respiration ratio	**Hydrological stress (H)**	**1**	**5.71E + 01**	**5.71E + 01**	**35.7**	**<0.001**
	Pesticides (P)	1	3.69E + 01	3.69E + 01	23.1	**<**0.001
	H:P	1	1.19E + 01	1.19E + 01	7.5	0.015

Significant results for single and multiple stressors are presented (P-value < 0.05). Acronyms: H = hydrological stress, P = pesticides, W = warming, df = degrees of freedom, SS = sum of squares, MS = mean of squares, F = *F*-value, P = *P*-value. The strongest effect (i.e. highest F-value) for each response variable is highlighted in bold. Substrate utilization diversity does not appear in the table as none of the factors included in the ANOVA were significant (P > 0.05). For the complete ANOVA output, including non-significant effects and residuals, see supplementary information.

#### Single stressor responses (*main effects*)

Hydrological stress (H) applied as reduced flow produced the most severe effects in the river biofilms employed in this experiment (Figs 1 and 2), significantly altering 8 out of the 11 response variables assessed (Table 2). Hydrological stress significantly reduced total chlorophyll-a concentration (F_1,16_ = 57.6, P < 0.001, Fig. 1A), basal chlorophyll fluorescence (F_1,16_ = 107.4, P < 0.001, Fig. 1D), photosynthetic efficiency (F_1,16_ = 249.4, P < 0.001, Fig. 1B) and photosynthetic capacity (F_1,16_ = 61.1, P < 0.001, Fig. 1C). These effects were particularly intense for epilithic biofilm, making the interaction between hydrological stress and substratum type significant for most of the response variables assessed (Table S4). This translated into altered gross primary production in biofilms submitted to hydrological stress (Fig. S2; F_1,16_ = 30.1, P < 0.001). Water warming significantly altered 4 out of the 11 response variables assessed (Table 2). Warming slightly decreased photosynthetic capacity (Fig. 1C; F_1,16_ = 8.6, P = 0.010) and 16S rRNA gene abundance (Fig. 1F; F_1,16_ = 33.7, P < 0.001). Warming had an overall significant main effect on community respiration (F_1,16_ = 41.3, P < 0.001), although the levels of oxygen consumption in W treatment were comparable to those found on control streams. These effects on metabolic rates translated into altered production-respiration ratios (Fig. 2), with special impact of hydrological stress (F_1,16_ = 35.7, P < 0.001). Pesticides significantly altered 4 out of the 11 response variables (Table 2), namely photosynthetic efficiency (Fig. 1B; F_1,16_ = 50.7, P < 0.001) and metabolic rates (Figs 2, S2). The negative effects of pesticides on photosynthetic efficiency were more pronounced in the epilithic biofilm, making the interaction between pesticides and substratum type significant (Table S4).

#### Multiple stressor responses (*interactive effects*)

We assessed the effects of 4 different stressor combinations (i.e. W:H, W:P, H:P, W:H:P) on 11 response variables at 2 different time scales (short term vs. long term) and on 2 different substratum types (cobbles; epilithic and sand; epipsammic). Out of the 152 possible combinations, 108 resulted in non-significant interaction terms, suggesting additive effects (71%), whereas 44 resulted in significant interactions (29%). Detailed information on stressor combinations and interactive effects is available in Table S6. These interactions were mostly antagonistic, meaning that the combined effect of the stressors was less pronounced than the sum of the individual effects.

Chlorophyll-*a* concentration and photosynthetic efficiency antagonistically responded to the combination of hydrological stress and pesticides (H:P; F_1,16_ = 10.5 and 5.6, P = 0.005 and < 0.001, Table 2); when combined, these stressors resulted in chlorophyll-*a* concentration and photosynthetic efficiency values that were higher than the values that would be obtained assuming additive effects (Fig. 1A,B). In the case of chlorophyll-*a* concentration, this antagonistic interaction was only observed for epilithic biofilm, making the triple interaction between hydrological stress, pesticides and substratum type significant (Table S4). Antagonism was also observed for gross primary production (H:P; F_1,16_ = 13.8, P = 0.002), community respiration (H:P; F_1,16_ = 7.9, P = 0.013) and production-respiration ratios (H:P; F_1,16_ = 7.5, P = 0.015). The combination between warming and pesticides (W:P) resulted in an antagonistic interaction for basal chlorophyll fluorescence (F_1,16_ = 5.5, P = 0.033). The warming-pesticides combination also decreased production-respiration ratios, favoring heterotrophic conditions, although the interaction was only significant for gross primary production (F_1,16_ = 8.2, P = 0.011). The combination between warming and hydrological stress (W:H) mostly interacted to alter heterotrophic metabolism, as indicated by significant interaction terms for organic substrate utilization richness (F_1,16_ = 10.0, P = 0.006), and community respiration (F_1,16 = _6.0, P = 0.026). The combination between the three stressors (W:H:P) was significant for basal chlorophyll fluorescence (F_1,16_ = 5.1, P = 0.038) and community respiration (F_1,16_ = 8.1, P = 0.012).

#### Single and multiple stressor responses at short and long-term exposure times

Single stressor responses were highly dependent on exposure time (Table 3). The negative effects of hydrological stress (H) on primary producers were further amplified at long term, especially for photosynthetic capacity (H:T; F_1,16_ = 90.5, P < 0.001) and basal chlorophyll fluorescence (H:T; F_1,16_ = 108.5, P < 0.001). This was particularly the case for epilithic biofilm, as indicated by a significant triple interaction between hydrological stress, exposure time and substratum type (Table S5). The number of 16S rRNA gene copies dropped after long-term exposure to hydrological stress conditions especially in the epilithic biofilm (Fig. 1F). As observed for hydrological stress, pesticides produced their negative impact on production-respiration ratios only after long-term exposure (Fig. 2), making the interaction between pesticides and exposure time significant (F_1,16_ = 15.9, P = 0.001).

**Table 3:** Output for the mixed-model nested ANOVA (random factor *time*).

Response variable	Factor	df	SS	MS	F	P
Photosynthetic efficiency	**Time (T)**	**1**	**1.11E + 05**	**1.11E + 05**	**54.4**	**<0.001**
	H:T	1	3.04E + 04	3.04E + 04	14.9	0.001
	W:P:T	1	7.32E + 04	7.32E + 04	35.8	**<**0.001
Photosynthetic capacity	Time (T)	1	6.92E + 04	6.92E + 04	24.6	**<**0.001
	**H:T**	**1**	**2.54E + 05**	**2.54E + 05**	**90.5**	**<0.001**
Basal chlorophyll fluorescence	Time (T)	1	1.88E + 05	1.88E + 05	31.5	**<**0.001
	W:T	1	3.20E + 04	3.20E + 04	5.3	0.034
	**H:T**	**1**	**6.48E + 05**	**6.48E + 05**	**108.5**	**<0.001**
	W:P:T	1	8.00E + 04	8.00E + 04	13.4	0.002
	W:H:P:T	1	3.53E + 04	3.53E + 04	5.9	0.027
Leucine aminopeptidase activity	**Time (T)**	**1**	**2.89E + 04**	**2.89E + 04**	**109.4**	**<0.001**
	H:T	1	2.63E + 03	2.63E + 03	9.9	0.006
	P:T	1	1.44E + 03	1.44E + 03	5.4	0.033
16S rRNA gene abundance	**Time (T)**	**1**	**1.42E + 15**	**1.42E + 15**	**32.1**	**<0.001**
	W:T	1	2.26E + 14	2.26E + 14	5.1	0.038
Substrate utilization richness	**Time (T)**	**1**	**6.34E + 01**	**6.34E + 01**	**17.4**	**<0.001**
Substrate utilization diversity	**Time (T)**	**1**	**1.23E-01**	**1.23E-01**	**20.8**	**<0.001**
	P:T	1	3.05E-02	3.05E-02	5.1	0.037
Gross primary production	**Time (T)**	**1**	**4.40E + 01**	**4.40E + 01**	**104.2**	**<0.001**
	H:T	1	1.52E + 01	1.52E + 01	35.9	**<**0.001
Community respiration	Time (T)	1	5.89E + 03	5.89E + 03	54	**<**0.001
	**W:T**	**1**	**1.34E + 03**	**1.34E + 03**	**12.2**	**0.003**
	W:H:T	1	9.49E-01	9.49E-01	8.7	0.01
Production-respiration ratio	Time (T)	1	2.84E + 01	2.84E + 01	18.8	**<**0.001
	**H:T**	**1**	**4.65E + 01**	**4.65E + 01**	**30.8**	**<0.001**
	P:T	1	2.40E + 01	2.40E + 01	15.9	0.001
	H:P:T	1	1.12E + 01	1.12E + 01	7.4	0.015

Significant interactions with time are presented (P-value < 0.05). Acronyms: H = hydrological stress, P = pesticides, W = warming, df = degrees of freedom, SS = sum of squares, MS = mean of squares, F = *F*-value, P = *P*-value. The strongest effect (i.e. highest F-value) for each response variable is highlighted in bold. Chlorophyll-*a* concentr*a*tion does not appear in the table as none of the factors included in the ANOVA were significant (P > 0.05). For the complete ANOVA output, including non-significant effects and residuals, see supplementary information.

Overall, exposure time promoted antagonistic interactions. At short term, the 59% of significant interactions were classified as antagonisms, and the 41% as synergisms. On the other hand, the 86% of the significant interactions were antagonisms at long term, and only the 14% were synergisms by then (Table S6). The interaction between warming and pesticides (W:P) was the most affected by exposure time (Table 3). The negative effects of warming and pesticides on photosynthetic efficiency and basal chlorophyll-*a* fluorescence were mitigated after long-term exposure to both stressors (F_1,16_ = 35.8, P < 0.001; F_1,16_ = 13.4, P = 0.002). Similarly, the response to warming and hydrological stress in terms of community respiration became antagonism at long term (W:H:T; F_1,16_ = 8.7, P = 0.010). Also in line with this, the little effect of the interaction between hydrological stress and pesticides on production-respiration ratios became antagonistic after long-term exposure to the same combination of stressors (H:P:T; F_1,16_ = 7.4, P = 0.015).

## Discussion

### Considerations on the experimental design and treatment conditions

Manipulative experiments in the laboratory can define causative relationships between stressors and the response to them^44^. These experiments can also provide insight on the mechanisms involved and the effects over exposure time^36,45^. Nevertheless, laboratory experiments are simplifications of the reality, as they replace the complexity of natural settings by only a few factors. In order to properly address the effects of Global change on ecosystems, manipulative experiments need to be able to reproduce natural communities under controlled conditions. The artificial streams used in this study were previously demonstrated to be able to reproduce up to 91.6% of the bacterial operational taxonomic units (OTUs) present in the original site (i.e. Llémena River), sharing a 72.7% Bray-Curtis similarity^40^. At the primary producers level, the artificial streams used in this study reproduce a typical river biofilm community, with dominance of diatoms (60% of total abundance) and lower proportions of Cyanobacteria (16%), Chlorophyta (13%) and Rhodophyta (7%)^46^. At the functional level, we measured photosynthetic efficiencies and organic matter degradation before any experimental manipulation (i.e. end of the acclimation phase), and we obtained results equivalent to those found for river biofilms in the Mediterranean region^38,47,48^. A detailed comparison between the biofilms used in this study and those developing under realistic environmental conditions is presented in Table S3.

Experimental conditions in control streams (i.e. light availability, water temperature, water velocity and/or available nutrients) favored the prevalence of the phototrophic community. Basal chlorophyll fluorescence, chlorophyll-*a* concentration, and production-respiration ratios therefore significantly increased with time in control streams, whereas the abundance of total bacteria (predicted from 16S rRNA gene abundance) decreased. This trend was especially evident for the epilithic biofilm, where significant interactions between time and substratum type occurred. Whereas the average nutrient concentrations in our artificial streams were low (especially for nitrite, ammonia and phosphate), water temperature, light and flow conditions may have promoted algal growth in our artificial streams.

Stressor levels used in our experiment represented realistic current values as well as estimates from Climate Change projections^8,9^. Appropriately selecting stressor levels is critical to avoid one or few factors dominating over the others^49^. So forth, the increase in water temperature we applied (see section 3.1) lies within the modeled projection of 0.3–4.8 °C increase in global mean surface temperature by the end of the 21^st^ century, relative to 1986–2005^8^. Also, the low-flow situation applied is aligned with future climate change projections^9^. We here applied a controlled low-flow situation on which surface flow is removed, but sub-surface flow remains. Finally, the mixture of pesticides represented a common combination in Mediterranean systems^50–52^. A comparison between the pesticide concentrations achieved in our artificial streams and realistic concentrations in agricultural rivers and streams is presented in Table S2.

### Single stressor effects

The largest main effect observed was caused by hydrological stress, which impedes resource acquisition through the limitation of organic matter and nutrient diffusion, combined with osmotic stress^53^; it negatively altered both biofilm community structure and function, leading to 73% of the response variables being significantly altered. This effect was especially relevant for epilithic biofilms, probably because of the low porosity of cobbles, which lead to severe desiccation and decreased productivity in biofilms subjected to hydrological stress. The negative effects of low-flow on the epilithic biofilm were observed at both short and long term, although the magnitude of the effect was higher after long-term exposures (i.e. 30 days). In this situation, the interaction between hydrological stress and time was significant for 54% of response variables. These results partially confirm our first hypothesis predicting that climatic stressors would cause the most pervasive effects on the biofilm.

The large impact of hydrological stress on the autotrophic compartment (i.e. affecting photosynthetic parameters) also had an indirect effect on heterotrophic functioning. We observed a decrease in the decomposition rate of peptidic compounds (measured as the leucine aminopeptidase activity, LAPA), suggesting decreased availability of primary producers-derived organic compounds. The effects on LAPA were smaller in the long term, suggesting that heterotrophic microorganisms in epilithic biofilms could become adapted to hydrological stress, shifting towards the use of other substrates, as indicated by the increase in substrate utilization diversity after 30 days of exposure.

A 1.6 °C increase in water temperature significantly altered a 36% of response variables, suggesting that water warming produce smaller effects than hydrological stress on river biofilms. Water temperature caused an overall positive effect in the 16S rRNA gene abundance, while it reduced the photosynthetic capacity of the epipsammic biofilm. So forth, temperature increase may have favored the bacterial heterotrophic capacity within the biofilms, confirmed by the reduction in gross primary production after long-term exposure to warming. Our observations match those of previous studies showing the positive effects of temperature on bacterial growth and organic carbon degradation^25,54^.

Finally, pesticides impaired mainly the autotrophic compartment, with a 36% of response variables being significantly altered. The reduction in photosynthetic efficiency was immediate in the epipsammic biofilm (i.e. after 48 hours), and only at long term (i.e. 30 days) could we observe reduced photosynthetic efficiency in the epilithic biofilm. This lower tolerance of epipsammic biofilms to pesticide exposure might be associated with the particularly high sensitivity of microorganisms in this biofilm^55^, as well as the increased porosity and retention capacity of fine sediments which facilitate the accumulation of toxicants, promoting biofilm exposure^56^.

### Multiple stressor effects

Biofilm responses to multiple stressors depend on the ability of its organisms to respond to each stressor and on the possible occurrence of positive or negative co-tolerance mechanisms^30,32^. An exposure to a stressor combined with a positive co-tolerance should reduce the impact of a second stressor, while a negative co-tolerance would have the adverse effect^57^. We here applied a null model comparison (as our null hypothesis predicted additive effects), and found that the majority of the stressor combinations (71%) did not result in significant interaction terms in the mixed-model nested ANOVA, suggesting the existence of overall additive effects. This prevalence of additive effects is consistent with previously published research, including analyses with higher statistical power^6,58,59^. However, we also found non-additive significant interactions (29% of all stressor combinations) regarding the three studied stressors (warming, hydrological stress and pesticides). We found that antagonisms prevailed among significant interactions and that exposure time lead the overall multiple-stressor response towards increased number of antagonistic interactions (from 59% of all significant interactions at short term to 86% at long term). The occurrence of these antagonistic effects agrees with previous research indicating that antagonisms are common at the community level in freshwaters^2,7^.

The antagonistic interaction between warming and pesticides (W:P) particularly occurred after long-term exposure; the negative individual effects of W and P on photosynthetic efficiency and basal chlorophyll fluorescence were partially mitigated. This antagonistic interaction was enhanced with exposure time, especially in the epilithic biofilm. Other studies have also shown partial mitigation of individual effects on river biofilms when warming and pesticides co-occur^26,60^. Warming and hydrological stress (W:H) resulted in antagonistic interactions concerning the heterotrophic activity. Organic substrate utilization richness and overall CR responded analogously. The organic substrate utilization richness in the epilithic biofilms for the W:H treatment reached values resembling more the controls than those under single-stressor treatments W and H. Strikingly, the W and H single-stressor treatments did not differed from the controls in terms of CR, but reached a 4.7-fold increase in oxygen consumption after 30 days of exposure when the two stressors co-occurred. In line with this, the lowest production-respiration ratios were recorded in the W:H streams, suggesting that this stressor combination promotes heterotrophy. The *ecological surprises* arising from the interaction between warming and hydrological stress have recently been highlighted on river biofilms^26^, and might be due to the metabolic activation of desiccation-tolerant taxa by temperature^61^.

Hydrological stress and pesticides (H:P) produced antagonistic interactions on both chlorophyll-*a* concentration and photosynthetic efficiency. This antagonism was especially relevant for epilithic biofilms after long-term exposure, which is probably related to the time lapse between the negative effects produced by hydrological stress (i.e. immediate) and those of pesticides (i.e. mostly after long-term exposure). Both hydrological stress and toxicant exposure have recently been reported to co-occur in 10–25% of rivers and transitional coastal waters worldwide^4^. The accumulation of extracellular polymeric substances (EPS) may lower the sensitivity of biofilms to organic chemicals^62,63^. As shown for monospecific biofilms^64^, the 30-day exposure to hydrological stress applied in this study could have favored the accumulation of EPS, hindering the penetration of the pesticides through the biofilm matrix. This antagonistic response was in line with production-respiration ratios, which decreased in the H:P treatment with respect to control streams, but less than expected based on individual H and P results.

Finally, the co-occurrence of warming, hydrological stress and pesticides (W:H:P) lead to the lowest values of photosynthetic efficiency, photosynthetic capacity and basal chlorophyll-*a* fluorescence in the epilithic biofilm. This interaction was however antagonistic for basal chlorophyll-*a* fluorescence, which showed levels above the additive prediction at long term. Overall, interactive effects among the three stressors (i.e. 3-way interactions) had smaller effect sizes (i.e. smaller computed F-values) than single stressors (i.e. main effects) and 2-way interactions. Interactions between 2 stressors drove the overall responses in our multiple stressors experiment, in a similar manner as indicated by other studies with higher statistical power^65–67^. The inclusion of climatic stressors (i.e. warming and hydrological stress) amongst the analyzed stressors is probably driving this pattern, as the W:H:P and the W:H combinations resulted in similar production-respiration ratios at long term, indicating little effects of pesticides in the triple interaction.

## Conclusions

Overall, our study reveals that river biofilms exposed to multiple global change stressors may partially adapt through changes in community structure and function, leading to antagonistic interactions, with combined effects that deviate from *a priori* predictions. Importantly, multiple stressor scenarios shifted the community metabolism towards heterotrophy, particularly when climatic stressors were at play. Ours study results may help mark the way forward for future studies assessing the nature of multiple stressor interactions across food webs in both artificial and natural settings.

## Supplementary information

Supplementary information

Supplementary dataset
