# Supplementary material for: Effects of multiple stressors on river biofilms depend on the time scale
Source: Sci Rep. 2019 Nov 1;9:15810. doi: 10.1038/s41598-019-52320-4 (PMC6825187; doi:10.1038/s41598-019-52320-4)
Supplement: Supplementary file 1 — Supplementary information [file 41598_2019_52320_MOESM1_ESM.pdf]

Effects of multiple stressors on river biofilms depend on the time scale

Supplementary Information

Ferran Romero[1,2]*, Vicenç Acuña[1,2], Carme Font[1,2], Anna Freixa[1,2], Sergi Sabater[1,3]

1 - Catalan Institute for Water Research (ICRA), C. Emili Grahit 101, 17003 Girona (Spain).

2 – Universitat de Girona (UdG), Girona (Spain)

3 - Institute of Aquatic Ecology (IEA), University of Girona, Campus de Montilivi, 17003 Girona (Spain).

* Corresponding author.

Author to whom correspondence should be addressed:

Ferran Romero

ICRA, Catalan Institute for Water Research

Emili Grahit 101, Building H_2O

Parc Científic i Tecnològic de la Universitat de Girona

E- 17003 Girona (Spain)

Tel: (+34) 972 18 33 80

Fax: (+34) 972 18 32 48

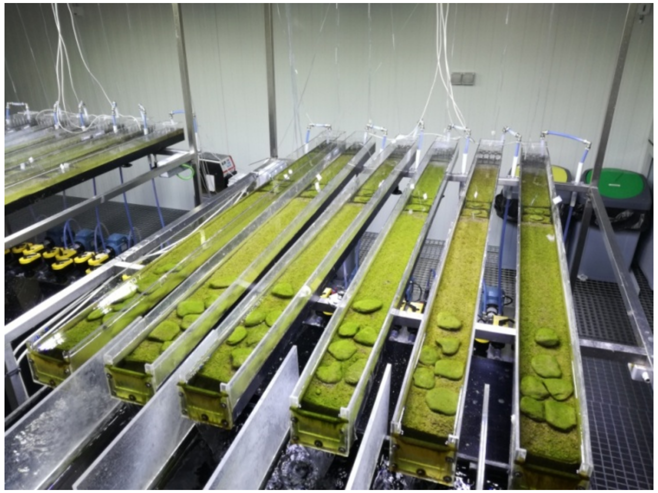

Fig. S1 Picture of 6 (out of 24) artificial streams used in this study installed at the Experimental Streams Facility. Each artificial stream consists of an independent methacrylate channel (l – w – d = 200 cm – 10 cm – 10 cm) and a 70 L water tank from which water can be recirculated.

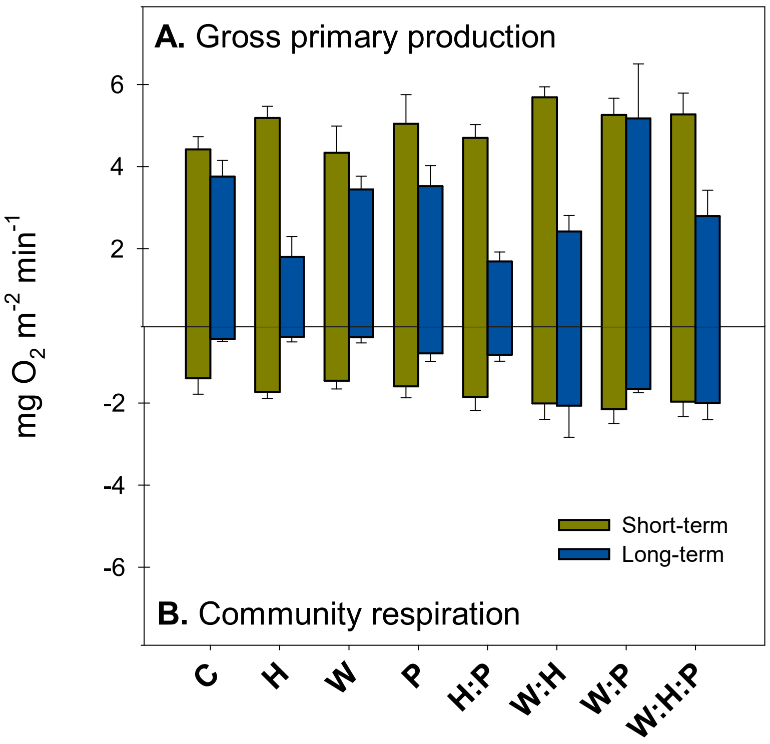

Fig. S2 Changes in metabolic rates (A; gross primary production, B; community respiration) for river biofilms after short and long-term exposure to the different treatments (H, W, P, H:P, W:H, W:P, W:H:P) and in control biofilms (C). Bars represent averaged values (n = 3), error bars represent standard errors (SE).

Pesticide	Target	Nominal conc. (ng L^{-1})	CAS number	Molecular weight (g mol^{-1})	Solubility (mg L^{-1})
Diuron	Herbicide	150	330 – 54 – 1	233.09	42.0
Chlorpyrifos	Insecticide	20	2921 – 88 – 2	350.59	1.4
Imazalil	Fungicide	120	35554 – 44 – 0	297.18	180.0
Prochloraz	Fungicide	30	67747 – 09 – 5	376.67	9.1
Simazine	Herbicide	50	122 – 34 – 9	201.66	5.0

Table S1 Pesticides employed in this experiment and nominal concentrations applied. Solubility is indicated for the given pesticides in water at 25° C.

Parameter	This study - artificial streams (influent water)	Field conditions			
		site	Season	value	reference
Diuron (ng L^{-1})	140.7 - 156.7	Zadorra River	A	150.96	[1]
Chlorpyrifos (ng L^{-1})	< 2.24	Ebro River	A	1.01 - 16.40	[1]
Imazalil (ng L^{-1})	15.1 - 85.4	Segre River	A	120	[1]
Prochloraz (ng L^{-1})	0.07 - 34.2	Segre River	A	34.2	[1]
Simazine (ng L^{-1})	50.3 - 68.6	Llobregat River	SP	53.6	[2]

Table S2 Comparison between pesticide concentrations used in this study and field conditions. All rivers in column "site" are located in the Iberian Peninsula (south of Europe). Abbreviations: A; autumn, SP; spring

Parameter	This study - biofilm before experimental manipulation (average ± SD , n = 24)		Field conditions				
	**Epilithic**	**Epipsammic**	**site**	**Biofilm type**	**Season**	**value**	**reference**
Chlorophyll-a concentration (μg cm^{-2})	11.05 ± 3.08	5.21 ± 0.18	Siurana River	Epilithic	A	8 - 10	[3, 4]
Photosynthetic efficiency (Y_{eff})	474 ± 60	373 ± 63	Cinca River	Epilithic	S	300 - 400	[3, 4]
Photosynthetic capacity (Y_{max})	630 ± 30	604 ± 42	Montsant River	Epilithic	A	500 - 600	[3, 4]
Leucine aminopeptidase activity (nmol AMC cm^{-2} h^{-1})	181 ± 31	26 ± 17	Cinca River	Epilithic	S	100 - 200	[3, 4]
EcoPlates - Substrate utilization diversity (Shannon's Index)	2.81 ± 0.10	2.87 ± 0.14	Llobregat River	Epipsammic	A	2.0 – 3.0	[5]

Table S3 Comparison between biofilm parameters before experimental manipulation (i.e. at the end of the acclimation period) and the same parameters in field conditions. Abbreviations: S; summer, W; winter, A; autumn, SP; spring

Response variable	Factor	df	SS	MS	F	P
Chlorophyll-a concentration	Substratum type (S)	1	1.01E+02	1.01E+02	20.5	< 0.001
	W:S	1	2.49E+01	2.49E+01	5.0	0.039
	H:S	1	3.20E+02	3.20E+02	65.2	< 0.001
	H:P:S	1	4.96E+01	4.96E+01	10.1	0.006
Photosynthetic efficiency	Substratum type (S)	1	4.09E+05	4.09E+05	298.4	< 0.001
	H:S	1	7.89E+05	7.89E+05	575.1	< 0.001
	P:S	1	1.69E+04	1.69E+04	12.3	0.003
Photosynthetic capacity	Substratum type (S)	1	6.92E+04	6.92E+04	24.6	< 0.001
	H:S	1	2.54E+05	2.54E+05	90.5	< 0.001
Basal chlorophyll-a fluorescence	Substratum type (S)	1	1.57E+05	1.57E+05	27.7	< 0.001
	H:S	1	3.79E+05	3.79E+05	66.7	< 0.001
Leucine aminopeptidase activity	Substratum type (S)	1	2.89E+04	2.89E+04	109.4	< 0.001
	H:S	1	2.63E+03	2.63E+03	9.9	0.006
	P:S	1	1.44E+03	1.44E+03	5.4	0.032
16S rRNA gene abundance	Substratum type (S)	1	2.52E+15	2.52E+15	33.6	< 0.001
	H:S	1	6.84E+14	6.84E+14	9.1	0.008
Substrate utilization richness	Substratum type (S)	1	5.70E+01	5.70E+01	9.3	0.007
Substrate utilization diversity	Substratum type (S)	1	7.65E-02	7.65E-02	10.1	0.006
	H:S	1	5.18E-02	5.18E-02	6.8	0.019
	P:S	1	6.05E-02	6.05E-02	8.0	0.012

Table S4 Output for the mixed-model nested ANOVA (random factor *substratum type*). Significant results for single and multiple stressors are presented (P-value < 0.05). Acronyms: S = substratum type, H = hydrological stress, P = pesticides, W = warming, df = degrees of freedom, SS = sum of squares, MS = mean of squares, F = *F*-value, P = *P*-value.

Response variable	Factor	df	SS	MS	F	P
Chlorophyll-a concentration	S:T	1	3.93E+02	3.93E+02	32.3	< 0.001
Photosynthetic efficiency	H:S:T	1	1.99E+04	1.99E+04	7.4	0.015
	W:H:P:S:T	1	2.99E+04	2.99E+04	11.1	0.004
Photosynthetic capacity	S:T	1	6.12E+05	6.12E+05	133.1	< 0.001
	H:S:T	1	3.78E+05	3.78E+05	82.2	< 0.001
Basal chlorophyll-a fluorescence	H:S:T	1	1.49E+05	1.49E+05	50.4	< 0.001
	W:P:S:T	1	1.99E+04	1.99E+04	6.8	0.019
Leucine aminopeptidase activity	S:T	1	2.34E+04	2.34E+04	16.5	< 0.001
16S rRNA gene abundance	S:T	1	2.88E+14	2.88E+14	7.6	0.013
	W:S:T	1	2.76E+14	2.76E+14	7.3	0.015
	W:H:P:S:T	1	2.36E+14	2.36E+14	6.3	0.023
Substrate utilization richness	S:T	1	5.70E+01	5.70E+01	5.9	0.027
Substrate utilization diversity	H:S:T	1	1.16E-01	1.16E-01	5.9	0.027

Table S5 Output for the mixed-model nested ANOVA (random factors S:T). Significant results for single and multiple stressors are presented (P-value < 0.05). Acronyms: T = time, S = substratum type, H = hydrological stress, P = pesticides, W = warming, df = degrees of freedom, SS = sum of squares, MS = mean of squares, F = *F*-value, P = *P*-value.

			W:H (T:S)	W:P (T:S)	H:P (T:S)	W:H:P (T:S)
Chlorophyll-a concentration	Short-term	epilithic	n.s.	n.s.	A	n.s.
		epipsammic	n.s.	n.s.	A	n.s.
	Long-term	epilithic	n.s.	n.s.	A	n.s.
		epipsammic	n.s.	n.s.	A	n.s.
Photosynthetic efficiency	Short-term	epilithic	n.s.	S	S	S
		epipsammic	n.s.	S	A	S
	Long-term	epilithic	n.s.	A	A	A
		epipsammic	n.s.	A	S	A
Photosynthetic capacity	Short-term	epilithic	n.s.	n.s.	n.s.	n.s.
		epipsammic	n.s.	n.s.	n.s.	n.s.
	Long-term	epilithic	n.s.	n.s.	n.s.	n.s.
		epipsammic	n.s.	n.s.	n.s.	n.s.
Basal chlorophyll fluorescence	Short-term	epilithic	n.s.	A	n.s.	A
		epipsammic	n.s.	A	n.s.	S
	Long-term	epilithic	n.s.	A	n.s.	A
		epipsammic	n.s.	A	n.s.	A
Leucine aminopeptidase activity	Short-term	epilithic	n.s.	n.s.	n.s.	n.s.
		epipsammic	n.s.	n.s.	n.s.	n.s.
	Long-term	epilithic	n.s.	n.s.	n.s.	n.s.
		epipsammic	n.s.	n.s.	n.s.	n.s.
16S rRNA gene abundance	Short-term	epilithic	n.s.	n.s.	n.s.	A
		epipsammic	n.s.	n.s.	n.s.	A
	Long-term	epilithic	n.s.	n.s.	n.s.	S
		epipsammic	n.s.	n.s.	n.s.	A
EcoPlates - substrate utilization richness	Short-term	epilithic	A	n.s.	n.s.	n.s.
		epipsammic	S	n.s.	n.s.	n.s.
	Long-term	epilithic	A	n.s.	n.s.	n.s.
		epipsammic	S	n.s.	n.s.	n.s.
EcoPlates - substrate utilization diversity	Short-term	epilithic	n.s.	n.s.	n.s.	n.s.
		epipsammic	n.s.	n.s.	n.s.	n.s.
	Long-term	epilithic	n.s.	n.s.	n.s.	n.s.
		epipsammic	n.s.	n.s.	n.s.	n.s.
Gross primary production	Short-term		n.s.	A	A	n.s.
	Long-term		n.s.	A	A	n.s.
Community respiration	Short-term		S	n.s.	A	A
	Long-term		A	n.s.	A	A
Production-respiration ratio	Short-term		n.s.	n.s.	S	n.s.
	Long-term		n.s.	n.s.	A	n.s.

Table S6 Stressor combinations assessed in this study (n = 152). Factors are: W; *warming*, H, *hydrological stress*, P; *pesticides*, T; *time* and S; *substratum type*. Parentheses indicate random (i.e. nested) factors. Out of the 152 stressor combinations, 44 (29 %) were significant at $P < 0.05$ (A; antagonism, S; synergism). Considering only short-term significant interactions (n = 22), 13 were classified as antagonisms (59 %), and 9 as synergisms (41 %). At long-term exposure (n = 22), 19 interactions were antagonistic (86 %) and 3 synergistic (14 %)

REFERENCES:

1. Ccanccapa A, Masiá A, Navarro-Ortega A, et al (2016) Pesticides in the Ebro River basin: Occurrence and risk assessment. Environ Pollut 211:414–424. https://doi.org/https://doi.org/10.1016/j.envpol.2015.12.059

2. Ricart M, Guasch H, Barceló D, et al (2010) Primary and complex stressors in polluted mediterranean rivers: Pesticide effects on biological communities. J Hydrol 383:52–61. https://doi.org/https://doi.org/10.1016/j.jhydrol.2009.08.014

3. Ponsatí L, Corcoll N, Petrovic M, et al (2016) Multiple-stressor effects on river biofilms under different hydrological conditions. Freshw Biol 61:2102–2115. https://doi.org/10.1111/fwb.12764

4. Ponsatí L, Acuña V, Aristi I, et al (2015) Biofilm Responses to Flow Regulation by Dams in Mediterranean Rivers. River Res Appl 31:1003–1016. https://doi.org/10.1002/rra.2807

5. Freixa A, Romaní AM (2014) Shifts in carbon substrate utilization in sediment microbial communities along the Llobregat River. Fundam Appl Limnol 18:247–262. https://doi.org/10.1127/fal/2014/0588
