# Supplementary material for: Effects of multiple stressors on river biofilms depend on the time scale
Source: Sci Rep. 2019 Nov 1;9:15810. doi: 10.1038/s41598-019-52320-4 (PMC6825187; doi:10.1038/s41598-019-52320-4)
Supplement: Supplementary file 2 — Supplementary dataset [file 41598_2019_52320_MOESM2_ESM.pdf]

Effects of multiple stressors on river biofilms depend on the time scale

Supplementary Dataset

ANOVA results (detailed)

Ferran Romero[1,2]*, Vicenç Acuña[1,2], Carme Font[1,2], Anna Freixa[1,2], Sergi Sabater[1,3]

1 - Catalan Institute for Water Research (ICRA), C. Emili Grahit 101, 17003 Girona (Spain).

2 – Universitat de Girona (UdG), Girona (Spain)

3 - Institute of Aquatic Ecology (IEA), University of Girona, Campus de Montilivi, 17003 Girona (Spain).

* Corresponding author.

Author to whom correspondence should be addressed:

Ferran Romero

ICRA, Catalan Institute for Water Research

Emili Grahit 101, Building H_2O

Parc Científic i Tecnològic de la Universitat de Girona

E- 17003 Girona (Spain)

Tel: (+34) 972 18 33 80

Fax: (+34) 972 18 32 48

Chlorophyll-a concentration ANOVA

FIXED FACTORS

	Df	SS	MS	F-value	P-value	
W	1	17.62	17.62	3.612	0.0755	.
H	1	280.95	280.95	57.586	1.09e-06	***
P	1	11.39	11.39	2.335	0.1460	
W:H	1	0.47	0.47	0.096	0.7605	
W:P	1	6.83	6.83	1.400	0.2540	
H:P	1	51.29	51.29	10.513	0.0051	**
W:H:P	1	10.42	10.42	2.136	0.1632	
Residuals	16	78.06	4.88			

Signif. codes: 0 ‘***’ 0.001 ‘**’ 0.01 ‘*’ 0.05 ‘.’ 0.1 ‘ ’ 1

REPEATED MEASURES (TIME)

	Df	SS	MS	F-value	P-value
Time	1	18.10	18.105	1.496	0.239
W:Time	1	22.15	22.147	1.830	0.195
H:Time	1	9.49	9.494	0.785	0.389
P:Time	1	4.86	4.856	0.401	0.535
W:H:Time	1	0.01	0.008	0.001	0.979
W:P:Time	1	0.50	0.503	0.042	0.841
H:P:Time	1	0.09	0.092	0.008	0.932
W:H:P:Time	1	1.52	1.518	0.125	0.728
Residuals	16	193.62	12.101		

SUBSTRATUM TYPE

	Df	SS	MS	F-value	P-value	
S	1	100.6	100.6	20.542	0.00034	***
W:S	1	24.9	24.9	5.082	0.03854	*
H:S	1	319.6	319.6	65.228	4.9e-07	***
P:S	1	18.2	18.2	3.713	0.07193	.
W:H:S	1	0.1	0.1	0.028	0.86988	
W:P:S	1	8.5	8.5	1.740	0.20566	
H:P:S	1	49.6	49.6	10.114	0.00581	**
W:H:P:S	1	16.2	16.2	3.306	0.08781	.
Residuals	16	78.4	4.9			

Signif. codes: 0 ‘***’ 0.001 ‘**’ 0.01 ‘*’ 0.05 ‘.’ 0.1 ‘ ’ 1

TIME * SUBSTRATUM TYPE

	Df	SS	MS	F-value	P-value	
S:Time	1	393.3	393.3	32.283	3.4e-05	***
W:S:Time	1	13.6	13.6	1.112	0.307	
H:S:Time	1	16.6	16.6	1.359	0.261	
P:S:Time	1	5.9	5.9	0.487	0.495	
W:H:S:Time	1	1.2	1.2	0.096	0.760	
W:P:S:Time	1	0.1	0.1	0.012	0.916	
H:P:S:Time	1	0.8	0.8	0.067	0.799	

W:H:P:S:Time	1	3.4	3.4	0.277	0.606
Residuals	16	194.9	12.2		

Signif. codes: 0 '***' 0.001 '**' 0.01 '*' 0.05 '.' 0.1 ' ' 1

Photosynthetic efficiency ANOVA

FIXED FACTORS

	Df	Sum Sq	Mean Sq	F-value	p-value	
W	1	148	148	0.054	0.8184	
H	1	7E+05	7E+05	249.436	3.52e-11	***
P	1	1E+05	1E+05	50.710	2.43e-06	***
W:H	1	298	298	0.110	0.7446	
W:P	1	3863	3863	1.427	0.2497	
H:P	1	15075	15075	5.566	0.0314	*
W:H:P	1	811	811	0.299	0.5918	
Residuals	16	43332	2708			

Signif. codes: 0 '***' 0.001 '**' 0.01 '*' 0.05 '.' 0.1 ' ' 1

REPEATED MEASURES (TIME)

	Df	Sum Sq	Mean Sq	F-value	p-value	
Time	1	1E+05	1E+05	54.443	1.56e-06	***
W:Time	1	7193	7193	3.527	0.07874	.
H:Time	1	30424	30424	14.916	0.00138	**
P:Time	1	3889	3889	1.907	0.18633	
W:H:Time	1	291	291	0.142	0.71083	
W:P:Time	1	73206	73206	35.892	1.88e-05	***
H:P:Time	1	83	83	0.040	0.84313	
W:H:P:Time	1	3688	3688	1.808	0.19750	
Residuals	16	32634	2040			

Signif. codes: 0 '***' 0.001 '**' 0.01 '*' 0.05 '.' 0.1 ' ' 1

SUBSTRATUM TYPE

	Df	Sum Sq	Mean Sq	F-value	p-value	
S	1	4E+05	4E+05	298.440	9.03e-12	***
W:S	1	1128	1128	0.822	0.37806	
H:S	1	8E+05	8E+05	575.173	5.72e-14	***
P:S	1	16934	16934	12.345	0.00288	**
W:H:S	1	4148	4148	3.024	0.10126	
W:P:S	1	491	491	0.358	0.55822	
H:P:S	1	4280	4280	3.120	0.09640	.
W:H:P:S	1	2	2	0.002	0.96754	
Residuals	16	21948	1372			

Signif. codes: 0 '***' 0.001 '**' 0.01 '*' 0.05 '.' 0.1 ' ' 1

TIME * SUBSTRATUM TYPE

	Df	Sum Sq	Mean Sq	F-value	p-value	
S:Time	1	8270	8270	3.061	0.09934	.
W:S:Time	1	357	357	0.132	0.72116	
H:S:Time	1	19924	19924	7.375	0.01527	*
P:S:Time	1	1060	1060	0.392	0.53989	
W:H:S:Time	1	3186	3186	1.179	0.29362	

W:P:S:Time	1	7263	7263	2.688	0.12059							
H:P:S:Time	1	250	250	0.093	0.76478							
W:H:P:S:Time	1	29857	29857	11.052	0.00429	**						
Residuals	16	43225	2702									

Signif.	codes:	0	‘***’	0.001	‘**’	0.01	‘*’	0.05	‘.’	0.1	‘ ’	1

Photosynthetic capacity ANOVA

FIXED FACTORS

	Df	Sum Sq	Mean S	F-value	p-value	
W	1	34884	34884	8.585	0.00981	**
H	1	2E+05	2E+05	61.097	7.48e-07	***
P	1	1944	1944	0.478	0.49906	
W:H	1	1204	1204	0.296	0.59370	
W:P	1	77	77	0.019	0.89220	
H:P	1	0	0	0.000	0.99245	
W:H:P	1	14900	14900	3.667	0.07356	.
Residuals	16	65017	4064			

Signif. codes: 0 ‘***’ 0.001 ‘**’ 0.01 ‘*’ 0.05 ‘.’ 0.1 ‘ ’ 1

REPEATED MEASURES (TIME)

	Df	Sum Sq	Mean S	F-value	p-value	
Time	1	69230	69230	24.664	0.00014	***
W:Time	1	963	963	0.343	0.56630	
H:Time	1	3E+05	3E+05	90.561	5.45e-08	***
P:Time	1	1365	1365	0.486	0.49559	
W:H:Time	1	2501	2501	0.891	0.35924	
W:P:Time	1	7633	7633	2.719	0.11864	
H:P:Time	1	451	451	0.161	0.69395	
W:H:P:Time	1	6370	6370	2.269	0.15144	
Residuals	16	44912	2807			

Signif. codes: 0 ‘*’** 0.001 ‘**’ 0.01 ‘*’ 0.05 ‘.’ 0.1 ‘ ’ 1

SUBSTRATUM TYPE

	Df	Sum Sq	Mean S	F-value	p-value	
S	1	69230	69230	24.664	0.00014	***
W:S	1	963	963	0.343	0.56630	
H:S	1	3E+05	3E+05	90.561	5.45e-08	***
P:S	1	1365	1365	0.486	0.49559	
W:H:S	1	2501	2501	0.891	0.35924	
W:P:S	1	7633	7633	2.719	0.11864	
H:P:S	1	451	451	0.161	0.69395	
W:H:P:S	1	6370	6370	2.269	0.15144	
Residuals	16	44912	2807			

Signif. codes: 0 ‘***’ 0.001 ‘**’ 0.01 ‘*’ 0.05 ‘.’ 0.1 ‘ ’ 1

TIME * SUBSTRATUM TYPE

	Df	Sum Sq	Mean S	F-value	p-value	
S:Time	1	6E+05	6E+05	133.121	3.63e-09	***
W:S:Time	1	51	51	0.011	0.917	
H:S:Time	1	4E+05	4E+05	82.190	1.06e-07	***
P:S:Time	1	3314	3314	0.721	0.408	
W:H:S:Time	1	17	17	0.004	0.953	

W:P:S:Time	1	13776	13776	2.997	0.103
H:P:S:Time	1	1785	1785	0.388	0.542
W:H:P:S:Time	1	3267	3267	0.711	0.412
Residuals	16	73538	4596		

Signif. codes: 0 ‘***’ 0.001 ‘**’ 0.01 ‘*’ 0.05 ‘.’ 0.1 ‘ ’ 1

Leucine aminopeptidase ANOVA

FIXED FACTORS

	Df	Sum Sq	Mean S	F-value	p-value	
W	1	2035	2035	1.558	0.22987	
H	1	13301	13301	10.185	0.00568	**
P	1	140	140	0.107	0.74745	
W:H	1	1395	1395	1.069	0.31665	
W:P	1	150	150	0.115	0.73908	
H:P	1	384	384	0.294	0.59511	
W:H:P	1	2521	2521	1.931	0.18370	
Residuals	16	20894	1306			

Signif. codes: 0 ‘***’ 0.001 ‘**’ 0.01 ‘*’ 0.05 ‘.’ 0.1 ‘ ’ 1

REPEATED MEASURES (TIME)

	Df	Sum Sq	Mean S	F-value	p-value	
Time	1	28912	28912	109.485	1.46e-08	***
W:Time	1	35	35	0.133	0.72042	
H:Time	1	2625	2625	9.941	0.00616	**
P:Time	1	1442	1442	5.459	0.03281	*
W:H:Time	1	92	92	0.349	0.56318	
W:P:Time	1	0	0	0.000	1.00000	
H:P:Time	1	122	122	0.460	0.50727	
W:H:P:Time	1	241	241	0.911	0.35395	
Residuals	16	4225	264			

Signif. codes: 0 ‘*’** 0.001 ‘**’ 0.01 ‘*’ 0.05 ‘.’ 0.1 ‘ ’ 1

SUBSTRATUM TYPE

	Df	Sum Sq	Mean S	F-value	p-value	
S	1	28912	28912	109.485	1.46e-08	***
W:S	1	35	35	0.133	0.72042	
H:S	1	2625	2625	9.941	0.00616	**
P:S	1	1441	1441	5.459	0.03281	*
W:H:S	1	92	92	0.349	0.56318	
W:P:S	1	0	0	0.000	1.00000	
H:P:S	1	121	121	0.460	0.50727	
W:H:P:S	1	241	241	0.911	0.35395	
Residuals	16	4225	264			

Signif. codes: 0 ‘***’ 0.001 ‘**’ 0.01 ‘*’ 0.05 ‘.’ 0.1 ‘ ’ 1

TIME * SUBSTRATUM TYPE

	Df	Sum Sq	Mean S	F-value	p-value	
S:Time	1	23375	23375	16.541	0.000896	***
W:S:Time	1	2	2	0.001	0.970150	
H:S:Time	1	1488	1488	1.053	0.320031	
P:S:Time	1	1667	1667	1.179	0.293571	
W:H:S:Time	1	1162	1162	0.822	0.377963	

W:P:S:Time	1	1040	1040	0.736	0.403595							
H:P:S:Time	1	620	620	0.439	0.517107							
W:H:P:S:Time	1	3174	3174	2.246	0.153427							
Residuals	16	22610	1413									

Signif.	codes:	0	‘***’	0.001	‘**’	0.01	‘*’	0.05	‘.’	0.1	‘ ’	1

Basal chlorophyll fluorescence ANOVA

FIXED FACTORS

	Df	Sum Sq	Mean S	F-value	p-value	
W	1	2017	2017	0.203	0.6581	
H	1	1E+06	1E+06	107.383	1.67e-08	***
P	1	459	459	0.046	0.8323	
W:H	1	7	7	0.001	0.9791	
W:P	1	54150	54150	5.459	0.0328	*
H:P	1	6734	6734	0.679	0.4221	
W:H:P	1	50876	50876	5.129	0.0378	*
Residuals	16	2E+05	9919			

Signif. codes: 0 '***' 0.001 '**' 0.01 '*' 0.05 '.' 0.1 ' ' 1

REPEATED MEASURES (TIME)

	Df	Sum Sq	Mean S	F-value	p-value	
Time	1	2E+05	2E+05	31.509	3.89e-05	***
W:Time	1	31974	31974	5.354	0.03429	*
H:Time	1	6E+05	6E+05	108.539	1.55e-08	***
P:Time	1	35	35	0.006	0.93989	
W:H:Time	1	975	975	0.163	0.69145	
W:P:Time	1	80041	80041	13.404	0.00211	**
H:P:Time	1	451	451	0.075	0.78705	
W:H:P:Time	1	35343	35343	5.919	0.02709	*
Residuals	16	95543	5971			

Signif. codes: 0 '*'** 0.001 '**' 0.01 '*' 0.05 '.' 0.1 ' ' 1

SUBSTRATUM TYPE

	Df	Sum Sq	Mean S	F-value	p-value	
S	1	2E+05	2E+05	27.710	7.72e-05	***
W:S	1	40	40	0.007	0.934	
H:S	1	4E+05	4E+05	66.790	4.20e-07	***
P:S	1	1944	1944	0.343	0.566	
W:H:S	1	1908	1908	0.336	0.570	
W:P:S	1	8177	8177	1.442	0.247	
H:P:S	1	425	425	0.075	0.788	
W:H:P:S	1	840	840	0.148	0.705	
Residuals	16	90735	5671			

Signif. codes: 0 '***' 0.001 '**' 0.01 '*' 0.05 '.' 0.1 ' ' 1

TIME * SUBSTRATUM TYPE

	Df	Sum Sq	Mean S	F-value	p-value	
S:Time	1	104	104	0.035	0.8532	
W:S:Time	1	5551	5551	1.883	0.1889	
H:S:Time	1	1E+05	1E+05	50.446	2.5e-06	***
P:S:Time	1	104	104	0.035	0.8532	
W:H:S:Time	1	368	368	0.125	0.7284	

W:P:S:Time	1	19895	19895	6.750	0.0194	*
H:P:S:Time	1	7245	7245	2.458	0.1365	
W:H:P:S:Time	1	1803	1803	0.612	0.4456	
Residuals	16	47157	2947			

Signif. codes: 0 ‘***’ 0.001 ‘**’ 0.01 ‘*’ 0.05 ‘.’ 0.1 ‘ ’ 1

16S rRNA gene abundance ANOVA

FIXED FACTORS

	Df	Sum Sq	Mean S	F-value	p-value	
W	1	1.558e+	1.558e+	33.740	2.67e-05	***
H	1	8.085e+	8.085e+	0.018	0.896	
P	1	3.824e+	3.824e+	0.828	0.376	
W:H	1	2.353e+	2.353e+	0.051	0.824	
W:P	1	3.059e+	3.059e+	0.662	0.428	
H:P	1	1.197e+	1.197e+	2.592	0.127	
W:H:P	1	2.391e+	2.391e+	0.052	0.823	
Residuals	16	7.389e+	4.618e+13			

Signif. codes: 0 '***' 0.001 '**' 0.01 '*' 0.05 '.' 0.1 ' ' 1

REPEATED MEASURES (TIME)

	Df	Sum Sq	Mean S	F-value	p-value	
Time	1	1.415e+	1.415e+	32.143	3.48e-05	***
W:Time	1	2.258e+	2.258e+	5.128	0.0378	*
H:Time	1	1.452e+	1.452e+	3.297	0.0882	.
P:Time	1	2.341e+	2.341e+	0.532	0.4764	
W:H:Time	1	2.580e+	2.580e+	0.586	0.4551	
W:P:Time	1	1.585e+	1.585e+	3.601	0.0759	.
H:P:Time	1	1.199e+	1.199e+	0.272	0.6089	
W:H:P:Time	1	2.281e+	2.281e+	0.518	0.4821	
Residuals	16	7.045e+	4.403e+13			

Signif. codes: 0 '*'** 0.001 '**' 0.01 '*' 0.05 '.' 0.1 ' ' 1

SUBSTRATUM TYPE

	Df	Sum Sq	Mean S	F-value	p-value	
S	1	2.515e+	2.515e+	33.622	2.72e-05	***
W:S	1	5.543e+	5.543e+	0.741	0.40208	
H:S	1	6.838e+	6.838e+	9.139	0.00808	**
P:S	1	1.704e+	1.704e+	0.023	0.88193	
W:H:S	1	6.939e+	6.939e+	0.093	0.76464	
W:P:S	1	3.672e+	3.672e+	0.491	0.49365	
H:P:S	1	8.811e+	8.811e+	1.178	0.29391	
W:H:P:S	1	3.094e+	3.094e+	0.004	0.94952	
Residuals	16	1.197e+	7.482e+13			

Signif. codes: 0 '***' 0.001 '**' 0.01 '*' 0.05 '.' 0.1 ' ' 1

TIME * SUBSTRATUM TYPE

	Df	Sum Sq	Mean S	F-value	p-value	
S:Time	1	2.878e+	2.878e+	7.646	0.0138	*
W:S:Time	1	2.755e+	2.755e+	7.318	0.0156	*
H:S:Time	1	6.894e+	6.894e+	1.831	0.1948	
P:S:Time	1	1.224e+	1.224e+	3.251	0.0903	.
W:H:S:Time	1	6.688e+	6.688e+	1.777	0.2012	

W:P:S:Time	1	3.756e+	3.756e+	0.100	0.7562							
H:P:S:Time	1	1.336e+	1.336e+	3.547	0.0779	.						
W:H:P:S:Time	1	2.362e+	2.362e+	6.275	0.0234	*						
Residuals	16	6.024e+	3.765e+13									

Signif.	codes:	0	‘***’	0.001	‘**’	0.01	‘*’	0.05	‘.’	0.1	‘ ’	1

BIOLOG Ecoplates substrate utilization richness ANOVA

FIXED FACTORS

	Df	Sum Sq	Mean S	F-value	p-value	
W	1	0.04	0.04	0.005	0.94502	
H	1	5.04	5.04	0.594	0.45216	
P	1	18.37	18.37	2.164	0.16063	
W:H	1	84.37	84.37	9.939	0.00616	**
W:P	1	0.38	0.38	0.044	0.83619	
H:P	1	0.04	0.04	0.005	0.94502	
W:H:P	1	35.04	35.04	4.128	0.05914	.
Residuals	16	135.83	8.49			

Signif. codes: 0 ‘***’ 0.001 ‘**’ 0.01 ‘*’ 0.05 ‘.’ 0.1 ‘ ’ 1

REPEATED MEASURES (TIME)

	Df	Sum Sq	Mean S	F-value	p-value	
Time	1	63.37	63.37	17.433	0.000715	***
W:Time	1	12.04	12.04	3.312	0.087521	.
H:Time	1	1.04	1.04	0.287	0.599813	
P:Time	1	12.04	12.04	3.312	0.087521	.
W:H:Time	1	3.38	3.38	0.928	0.349625	
W:P:Time	1	0.04	0.04	0.011	0.916074	
H:P:Time	1	1.04	1.04	0.287	0.599813	
W:H:P:Time	1	3.38	3.38	0.928	0.349625	
Residuals	16	58.17	3.64			

Signif. codes: 0 ‘*’** 0.001 ‘**’ 0.01 ‘*’ 0.05 ‘.’ 0.1 ‘ ’ 1

SUBSTRATUM TYPE

	Df	Sum Sq	Mean S	F-value	p-value	
S	1	57.04	57.04	9.393	0.00741	**
W:S	1	0.38	0.38	0.062	0.80691	
H:S	1	18.38	18.38	3.026	0.10115	
P:S	1	1.04	1.04	0.172	0.68426	
W:H:S	1	2.04	2.04	0.336	0.57011	
W:P:S	1	1.04	1.04	0.172	0.68426	
H:P:S	1	18.38	18.38	3.026	0.10115	
W:H:P:S	1	2.04	2.04	0.336	0.57011	
Residuals	16	97.17	6.07			

Signif. codes: 0 ‘***’ 0.001 ‘**’ 0.01 ‘*’ 0.05 ‘.’ 0.1 ‘ ’ 1

TIME * SUBSTRATUM TYPE

	Df	Sum Sq	Mean S	F-value	p-value	
S:Time	1	57.04	57.04	5.895	0.0274	*
W:S:Time	1	7.04	7.04	0.728	0.4062	
H:S:Time	1	35.04	35.04	3.621	0.0752	.
P:S:Time	1	9.37	9.37	0.969	0.3396	
W:H:S:Time	1	2.04	2.04	0.211	0.6522	

W:P:S:Time	1	1.04	1.04	0.108	0.7471
H:P:S:Time	1	1.04	1.04	0.108	0.7471
W:H:P:S:Time	1	12.04	12.04	1.244	0.2811
Residuals	16	154.83	9.68		

Signif. codes: 0 ‘***’ 0.001 ‘**’ 0.01 ‘*’ 0.05 ‘.’ 0.1 ‘ ’ 1

BIOLOG Ecoplates substrate utilization diversity ANOVA

FIXED FACTORS

	Df	Sum Sq	Mean S	F-value	p-value	
W	1	0.0213	0.02130	0.872	0.3642	
H	1	0.0008	0.00076	0.031	0.8622	
P	1	0.0699	0.06988	2.861	0.1101	
W:H	1	0.0944	0.09438	3.864	0.0669	.
W:P	1	0.0053	0.00525	0.215	0.6491	
H:P	1	0.0010	0.00100	0.041	0.8421	
W:H:P	1	0.0153	0.01525	0.624	0.4409	
Residuals	16	0.3908	0.02442			

\---

Signif. codes: 0 ‘***’ 0.001 ‘**’ 0.01 ‘*’ 0.05 ‘.’ 0.1 ‘ ’ 1

REPEATED MEASURES (TIME)

	Df	Sum Sq	Mean S	F-value	p-value	
Time	1	0.12255	0.12255	20.819	0.000319	***
W:Time	1	0.02130	0.02130	3.619	0.075293	.
H:Time	1	0.00008	0.00008	0.014	0.906193	
P:Time	1	0.03046	0.03046	5.174	0.037036	*
W:H:Time	1	0.00496	0.00496	0.843	0.372311	
W:P:Time	1	0.00863	0.00863	1.465	0.243652	
H:P:Time	1	0.01475	0.01475	2.506	0.132982	
W:H:P:Time	1	0.00586	0.00586	0.995	0.333277	
Residuals	16	0.09418	0.00589			

\---

Signif. codes: 0 ‘***’ 0.001 ‘**’ 0.01 ‘*’ 0.05 ‘.’ 0.1 ‘ ’ 1

SUBSTRATUM TYPE

	Df	Sum Sq	Mean S	F-value	p-value	
S	1	0.07650	0.07650	10.117	0.00581	**
W:S	1	0.00158	0.00158	0.210	0.65329	
H:S	1	0.05180	0.05180	6.851	0.01867	*
P:S	1	0.06050	0.06050	8.001	0.01210	*
W:H:S	1	0.02313	0.02313	3.058	0.09948	.
W:P:S	1	0.01955	0.01955	2.586	0.12739	
H:P:S	1	0.02768	0.02768	3.660	0.07380	.
W:H:P:S	1	0.00005	0.00005	0.007	0.93554	
Residuals	16	0.12098	0.00756			

\---

Signif. codes: 0 ‘***’ 0.001 ‘**’ 0.01 ‘*’ 0.05 ‘.’ 0.1 ‘ ’ 1

TIME * SUBSTRATUM TYPE

	Df	Sum Sq	Mean S	F-value	p-value	
S:Time	1	0.03721	0.03721	1.900	0.1871	
W:S:Time	1	0.01283	0.01283	0.655	0.4301	
H:S:Time	1	0.11551	0.11551	5.898	0.0273	*
P:S:Time	1	0.01898	0.01898	0.969	0.3395	
W:H:S:Time	1	0.00388	0.00388	0.198	0.6624	

W:P:S:Time	1	0.00618	0.00618	0.315	0.5822
H:P:S:Time	1	0.01788	0.01788	0.913	0.3536
W:H:P:S:Time	1	0.01576	0.01576	0.805	0.3830
Residuals	16	0.31335	0.01958		

Signif. codes: 0 ‘***’ 0.001 ‘**’ 0.01 ‘*’ 0.05 ‘.’ 0.1 ‘ ’ 1

Gross Primary Production ANOVA

FIXED FACTORS

	Df	Sum Sq	Mean S	F-value	p-value							
W	1	3.419	3.419	18.682	0.000526	***						
H	1	5.515	5.515	30.138	4.94e-05	***						
P	1	1.077	1.077	5.886	0.027458	*						
W:H	1	0.328	0.328	1.794	0.199112							
W:P	1	1.495	1.495	8.168	0.011393	*						
H:P	1	2.525	2.525	13.801	0.001882	**						
W:H:P	1	0.548	0.548	2.996	0.102696							
Residuals	16	2.928	0.183									

Signif.	codes:	0	‘***’	0.001	‘**’	0.01	‘*’	0.05	‘.’	0.1	‘ ’	1

REPEATED MEASURES (TIME)

	Df	Sum Sq	Mean S	F-value	p-value							
Time	1	43.95	43.95	104.201	2.06e-08	***						
W:Time	1	0.63	0.63	1.500	0.238							
H:Time	1	15.15	15.15	35.929	1.87e-05	***						
P:Time	1	0.23	0.23	0.548	0.470							
W:H:Time	1	0.06	0.06	0.148	0.706							
W:P:Time	1	0.81	0.81	1.929	0.184							
H:P:Time	1	0.27	0.27	0.644	0.434							
W:H:P:Time	1	0.29	0.29	0.695	0.417							
Residuals	16	6.75	0.42									

**Signif.**	**codes:**	**0**	**‘***’**	0.001	‘**’	0.01	‘*’	0.05	‘.’	0.1	‘ ’	1

Community Respiration ANOVA

FIXED FACTORS

	Df	Sum Sq	Mean S	F-value	p-value	
W	1	4.148	4.148	41.342	8.32e-06	***
H	1	1.654	1.654	16.485	0.000909	***
P	1	1.632	1.632	16.264	0.000963	***
W:H	1	0.601	0.601	5.988	0.026327	*
W:P	1	0.107	0.107	1.070	0.316323	
H:P	1	0.793	0.793	7.905	0.012537	*
W:H:P	1	0.809	0.809	8.060	0.011849	*
Residuals	16	1.605	0.100			

Signif. codes: 0 '***' 0.001 '**' 0.01 '*' 0.05 '.' 0.1 ' ' 1

REPEATED MEASURES (TIME)

	Df	Sum Sq	Mean S	F-value	p-value	
Time	1	5.887	5.887	54.058	1.63e-06	***
W:Time	1	1.337	1.337	12.274	0.00294	**
H:Time	1	0.199	0.199	1.827	0.19533	
P:Time	1	0.191	0.191	1.756	0.20370	
W:H:Time	1	0.949	0.949	8.716	0.00936	**
W:P:Time	1	0.001	0.001	0.008	0.92796	
H:P:Time	1	0.036	0.036	0.328	0.57463	
W:H:P:Time	1	0.105	0.105	0.968	0.33971	
Residuals	16	1.742	0.109			

Signif. codes: 0 '*'** 0.001 '**' 0.01 '*' 0.05 '.' 0.1 ' ' 1

Production-respiration ratio ANOVA

FIXED FACTORS

	Df	Sum Sq	Mean S	F-value	p-value	
W	1	6.66	6.66	4.165	0.058130	.
H	1	57.07	57.07	35.688	1.95e-05	***
P	1	36.93	36.93	23.090	0.000194	***
W:H	1	0.87	0.87	0.544	0.471600	
W:P	1	0.02	0.02	0.012	0.915894	
H:P	1	11.92	11.92	7.454	0.014826	*
W:H:P	1	6.42	6.42	4.017	0.062273	.
Residuals	16	25.59	1.60			

\---

Signif. codes: 0 ‘***’ 0.001 ‘**’ 0.01 ‘*’ 0.05 ‘.’ 0.1 ‘ ’ 1

REPEATED MEASURES (TIME)

	Df	Sum Sq	Mean S	F-value	p-value	
Time	1	28.37	28.37	18.828	0.000508	***
W:Time	1	2.85	2.85	1.893	0.187835	
H:Time	1	46.49	46.49	30.858	4.35e-05	***
P:Time	1	23.97	23.97	15.910	0.001057	**
W:H:Time	1	3.35	3.35	2.223	0.155400	
W:P:Time	1	0.06	0.06	0.041	0.842266	
H:P:Time	1	11.23	11.23	7.455	0.014817	*
W:H:P:Time	1	3.77	3.77	2.505	0.133038	
Residuals	16	24.11	1.51			

\---

Signif. codes: 0 ‘***’ 0.001 ‘**’ 0.01 ‘*’ 0.05 ‘.’ 0.1 ‘ ’ 1
